# Topologies, structures and parameter files for lipid simulations in GROMACS with the OPLS-aa force field: DPPC, POPC, DOPC, PEPC, and cholesterol

**DOI:** 10.1016/j.dib.2015.09.013

**Published:** 2015-09-26

**Authors:** Waldemar Kulig, Marta Pasenkiewicz-Gierula, Tomasz Róg

**Affiliations:** a Department of Physics, Tampere University of Technology, PO Box 692, FI-33101 Tampere, Finland; b Faculty of Biochemistry, Biophysics and Biotechnology, Jagiellonian University, ul. Gronostajowa 7, 30-387 Kraków, Poland

## Abstract

In this data article we provide topologies and force field parameters files for molecular dynamics simulations of lipids in the OPLS-aa force field using the GROMACS package. This is the first systematic parameterization of lipid molecules in this force field. Topologies are provided for four phosphatidylcholines: saturated DPPC, mono-cis unsaturated POPC and DOPC, and mono-trans unsaturated PEPC. Parameterization of the phosphatidylcholines was achieved in two steps: first, we supplemented the OPLS force field parameters for DPPC with new parameters for torsion angles and van der Waals parameters for the carbon and hydrogen atoms in the acyl chains, as well as new partial atomic charges and parameters for torsion angles in the phosphatidylcholine and glycerol moieties [1]. Next, we derived parameters for the cis and trans double bonds and the neighboring them single bonds [2]. Additionally, we provide GROMACS input files with parameters describing simulation conditions (md.mdp), which are strongly recommended to be used with these lipids models. The data are associated with the research article “Cis and trans unsaturated phosphatidylcholine bilayers: a molecular dynamics simulation study” [2] and provided as supporting materials.

**Specifications table**

**Table:** 

Subject area	*Chemistry*

More specific subject area	*Molecular dynamics simulations of lipid bilayers.*
Type of data	*GROMACS input files*
How data was acquired	Electronic structure and energy calculations were carried out using the GAUSSAN-03/09 suite following Hybrid Methods for Interaction Energies.
Data format	*text format*
Experimental factors	*Not applicable*
Experimental features	*Not applicable*
Data source location	*Not aplicable*
Data accessibility	*Data are supplied with this article*

## Value of the data

• New parameterization for lipids compatible with the OPLS-aa force field was derived.
• Provided files allow one to perform simulations of lipid bilayers with high accuracy.
• New lipids models are suitable for use with proteins parameterized in the OPLS-aa force field.

## 1 Data

Quality of molecular dynamics simulations ultimately depends on the accuracy of the force field parameters. Unfortunately derivation and validation of the force field for given classes of molecules is a tedious and lengthy process. Particularly time consuming is the derivation of torsional potential which is also a key factor for quality of molecular structure in classical simulation. Lipid bilayers are essential macromolecules and component of all living cells thus of high scientific interest also in modeling studies. High quality force fields for lipids started to be intensively developed during the last few years (for review see [3]). For example lipids models compatible with CHARMM force field which reproduced well properties of lipids bilayers were derived during last four years [4,5]. The AMBER force field model called Lipid 14 was delivered last year [6]. The third most popular force field, OPLS-AA [7,8] originally did not include lipids molecules. This gap was filled last year by our new model of saturated lipids [1] and now by a model of unsaturated lipids [2]. Data provided with this article consist of whole sets of force field parameters describing unsaturated lipids, compatible with OPLS force field.

## 2 Experimental design, materials and methods

### 2.1 Force field overview

New torsional parameters were calculated for the cis and trans double bonds and two subsequent single bonds in 3-decene and 5-decene. Bond stretching, angle bending, partial atomic charges, and Lennard–Jones parameters for sp^2^ carbon atoms were taken from the original OPLS-AA force field [7]. For sp^3^ carbon atoms, Lennard–Jones parameters derived for long saturated hydrocarbons in our previous study were used [1]. All quantum mechanics (QM) calculations were performed with GAUSSAN-03/09 [9] and molecular mechanics (MM) calculations were run in GROMACS 4.7 [10].

### 2.2 Procedure to derived torsion angle parameters

The total potential energy profile for rotation of a given dihedral angle was sampled by successive QM structure optimizations in vacuum. The QM energy was calculated for dihedral angles at 10° intervals. At each step, the remaining internal degrees of freedom were allowed to relax during the energy minimization procedure. A similar procedure was employed to perform molecular mechanics calculations with the torsion potential for the active coordinate set to zero. A restraining force constant was adjusted such that it allowed, at most, a ±1.5° deviation from the desired value of the sampled dihedral angle. Because restraints contribute to the evaluated potential energy, post-scans were needed to reevaluate the total potential energy without imposed restraints. As a result, the profile of the total potential energy, without an energetic contribution from the active dihedral, was derived. The functional form reproducing the profile adopted in OPLS-aa force field is the sum of the first five terms of the cosine power series, the so-called Ryckaert–Bellemans (RB) dihedral potential. The RB coefficients were found by minimizing the least-square function of the differences between the QM and MM total potential energies and the corresponding Ryckaert–Bellemans potential values.

### 2.3 Evaluation of the QM potential energy

The Hybrid Methods for Interaction Energies (HM-IE) method was used for accurate but efficient energy evaluation [11,12]. The calculations were performed in three steps. First, the geometry of the hydrocarbon molecule was optimized at MP2/cc-pVDZ level; the minimum energy obtained in this way is denoted as MP2/SBS (Small Basis Set). Second, two total energies for the optimized structure were calculated, one using CCSD(T)/DZ, denoted as CCSD(T)/SBS, and the other using MP2/cc-pVTZ, denoted as MP2/LBS (Large Basis Set). Finally, the required total potential energy was estimated using Eq. (1) in Ref. [11].

### 2.4 Files description

Provided data include topology files of four lipid species: DPPC (1,2-dipalmitoyl-*sn*-glycero-3-phosphocholine), POPC (1-palmitoyl-2-oleoyl-*sn*-glycero-3-phosphocholine), DOPC (1,2-dioleoyl-*sn*-glycero-3-ethylphosphocholine), PEPC (1-palmitoyl-2-elaidoyl-*sn*-glycero*-*3*-*phosphocholine) and cholesterol. The order of atoms in all molecules matches CHARMM-GUI membrane builder [13]. The format of provided topologies corresponds to the GROMACS simulation package. It is highly recommended to use the same model of nonbonded interactions as the one used in the parameterization procedure (see [1] and attached md.mdp). Also frequency of neighbor list update might affect calculation accuracy therefore should not be changed.

## Conflict of interest

The authors declare that there is no conflict of interest on any work published in this paper.
